# The Mycobacterial MsDps2 Protein Is a Nucleoid-Forming DNA Binding Protein Regulated by Sigma Factors σ^A^ and σ^B^

**DOI:** 10.1371/journal.pone.0008017

**Published:** 2009-11-30

**Authors:** Ramachandran Saraswathi, Rakhi Pait Chowdhury, Sunanda Margrett Williams, Payel Ghatak, Dipankar Chatterji

**Affiliations:** Molecular Biophysics Unit, Indian Institute of Science, Bangalore, India; University of Hyderabad, India

## Abstract

The Dps (DNA-binding protein from starved cells) proteins from *Mycobacterium smegmatis* MsDps1 and MsDps2 are both DNA-binding proteins with some differences. While MsDps1 has two oligomeric states, with one of them responsible for DNA binding, MsDps2 has only one DNA-binding oligomeric state. Both the proteins however, show iron-binding activity. The MsDps1 protein has been shown previously to be induced under conditions of starvation and osmotic stress and is regulated by the extra cellular sigma factors σ^H^ and σ^F^. We show here, that the second Dps homologue in *M. smegmatis*, namely MsDps2, is purified in a DNA-bound form and exhibits nucleoid-like structures under the atomic force microscope. It appears that the N-terminal sequence of Dps2 plays a role in nucleoid formation. MsDps2, unlike MsDps1, does not show elevated expression in nutritionally starved or stationary phase conditions; rather its promoter is recognized by RNA polymerase containing σ^A^ or σ^B^, under *in vitro* conditions. We propose that due to the nucleoid-condensing ability, the expression of MsDps2 is tightly regulated inside the cells.

## Introduction

Bacteria respond to stressful conditions encountered in the stationary phase of growth through a complex and intricate system of adaptation. This includes changes in metabolism, physiology as well as re-organization and protection of the cellular genetic material [1]. The methods of adaptation include the protection of the cellular genetic material, against physical and chemical assault, maintaining its integrity for subsequent growth and future generations. This is often accomplished by the re-organization of the genomic DNA and its compaction with the help of various single-strand DNA binding proteins and nucleoid proteins [2]. The DNA Binding Protein from Starved Cells, or Dps, is one such nucleoid-protein that is over expressed in *E. coli* under stationary phase conditions [3]. The Dps protein is mainly involved in the protection of the bacterial cell against oxidative stress. Lately, its role in the condensation and compaction of the bacterial genome in the stationary phase has been elucidated [4].

The first mycobacterial Dps protein was discovered in *Mycobacterium smegmatis* from a comparison of the protein profiles of well-nourished versus starved bacteria through proteomic analysis [5]. The MsDps1 protein was found to protect DNA against physical and chemical attack via its two oligomeric states, namely a trimer and a dodecamer [6]. Reassessment of structural stability under various pH conditions has been substantiated in other studies [7]. Further analysis indicated a tight regulation of expression of this protein *in vivo* with a conspicuous increase in expression in response to starvation and osmotic stress [8]. However, MsDps1, despite having a DNA binding ability in its dodecameric form, has not been associated with DNA-compaction activity so far. With the advent of a completely annotated *M. smegmatis* genome sequence in The Institute of Genome Research (www.tigr.org), a second Dps homologue, MsDps2, has been identified in *M. smegmatis.* Recently some of the structural and functional features of this new MsDps2 have been explored in comparison to MsDps1, based on crystal structure analysis and biochemical assays [9]. Structural analysis indicated a dodecameric conformation similar to MsDps1. However, the single oligomeric state and the ability to bind DNA in the absence of a characteristic DNA binding tail, as seen with MsDps1 [6], [10]–[12] suggested a unique function for MsDps2, distinct from that of MsDps1. We present here the evidence for the formation of MsDps2-DNA nucleoid like structure.

Interestingly, a promoter DNA-protein pull down experiment followed by single round *in vitro* transcription assay showed that RNA polymerase containing σ^A^ or σ^B^ is sufficient to carry out transcription at the *msdps2* promoter. This is different from the results we obtained in the case of the *msdps1* promoter [8], which is exclusively transcribed by extracytoplasmic function sigma factors. Thus, it raises the possibility that MsDps2 is tightly regulated, as a consequence of its ability for nucleoid formation within the cell.

## Results

### MsDps2 Protein Is Purified as a DNA-Dps Complex

**Figure 1:**
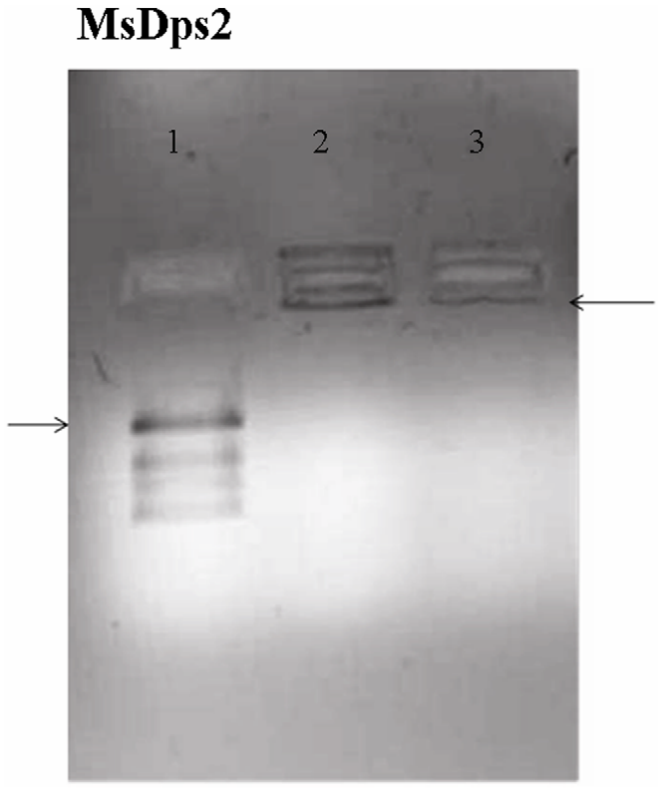
MsDps2 is purified as a DNA- bound complex. MsDps2 protein in 20 mM Tris-HCl (pH 7.9), 200 mM NaCl, purified by DE52 ion-exchange chromatography was checked on a 0.8% agarose gel for bound DNA. Lane 1: free plasmid DNA pGEM; lane 2: DNA+ MsDps2 (1∶10^4^ molar ratio); lane 3: purified MsDps2. The arrows indicate the position of the DNA entered in the agarose gel. Quantification of intensities of the DNA in the wells in Gel Retardation assay was done using Multi-Gauge V 2.3 (Fujifilm) software. The relative intensity/ (pixel)^ 2^ for DNA in samples of MsDps2 with respect to the blank (lane 1) were 12.14 for MsDps2 with DNA (lane 2) versus 8.28 (lane 3) for purified MsDps2 protein alone.

Figure 1 shows the DNA binding ability of the purified MsDps2 upon incubation with a plasmid DNA (pGEM plasmid 2.9 kb). It can be seen from the gel (lane 2) that the protein binds to DNA. This mode of binding for Dps proteins has been studied earlier, wherein the protein, upon DNA addition forms a huge protein-DNA complex that gets retained in the wells of an agarose gel [6], [9], [13], [14]. As the size of the complex is very big, we did not make any attempt to resolve the complex by other methods. Upon quantification of the band intensities using multigauge software, the amount of DNA was found to be more in lane 2, as compared to the free protein alone in lane 3 and values have been mentioned in the figure legend of figure 1. We inferred that even in lane 3 where no external DNA was added MsDps2 had DNA associated with it. Expectedly, as no added DNA was present, the intensity of DNA in lane 3 is less as compared to that in lane 2. Lane 1 containing the free DNA was used as control and therefore no DNA is present in the well. Further comparison of the DNA binding activity of the full length and deleted protein has been performed through AFM analysis and transmission electron microscopic studies as discussed below.

### MsDps2 Protein Forms Nucleoid-Like Structures *In Vitro*

The Dps proteins have a distinctive globular doughnut-like appearance with around 9 nm in diameter under the electron microscope and are known to form neatly organized two dimensional arrays *in vitro* [15].

**Figure 2:**
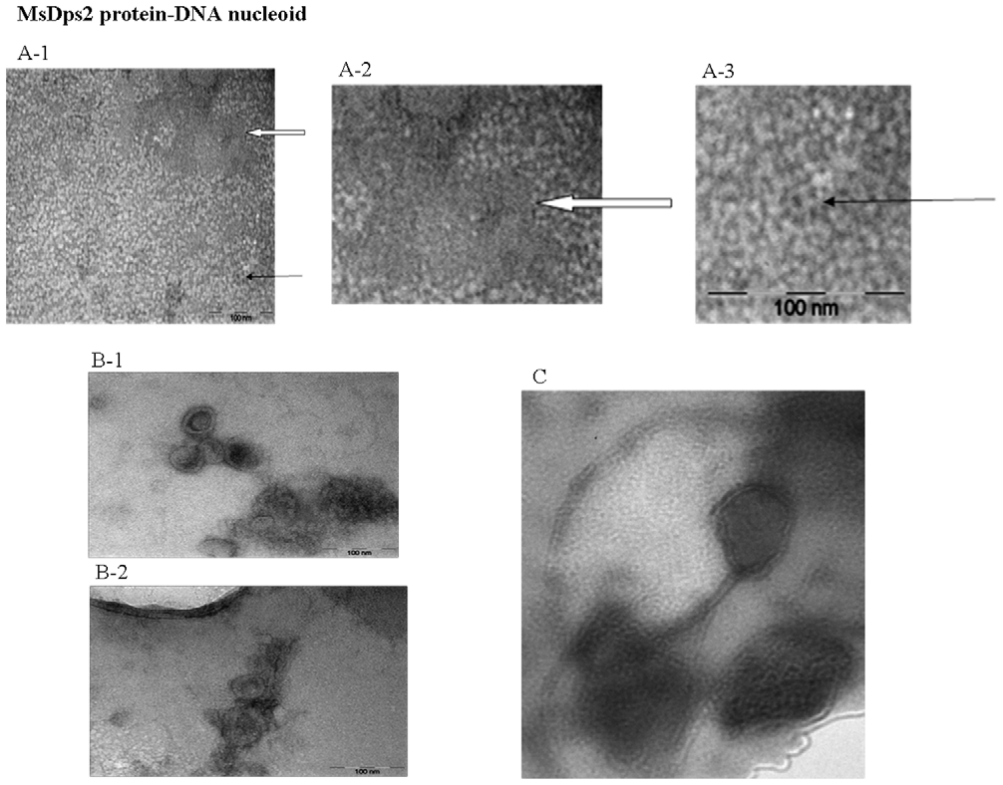
MsDps2 forms protein-DNA nucleoid-like structures under transmission electron microscope. A (1–3) MsDps2 forms ring-like structures and arrays. Purified MsDps2 protein shows ring-like doughnut-shaped dodecamers under the transmission electron microscope. A single particle is shown with the thin arrow. The protein was also seen to form arrays as shown in the region covered with the solid arrow. **B (1–2) MsDps2 protein forms nucleoid-like structures** ***in vitro*** **.** Transmission electron microscopic analysis of the MsDps2 protein bound to DNA showed nucleoid-like structures similar to those observed for the *E.coli* suggesting a role in the condensation and organization of the mycobacterial nucleoid for the MsDps2 protein. **C)** MsDps2 protein shows tightly compacted stationary phase nucleoid-like structure.

It was reported earlier that MsDps1 formed two dimensional arrays upon the addition of DNA to the protein. MsDps2 also forms the similar array in the presence of DNA under the transmission electron microscope in addition to the 9 nm diameter particle (Fig. 2).

In addition, we also found the presence of certain higher sized intricate structures of larger dimensions, made up of these globular proteins, under the electron microscope. The same was corroborated with the help of Atomic Force Microscopy and will be discussed in the following section. It has been reported earlier that the *E.coli* Dps protein forms nucleoid-like structures by coiling with DNA [15]. Dps is known to be involved in packaging the DNA to form compact bacterial nucleoid in the stationary phase, in addition to its primary role of protecting DNA under stress [4], [15]. The structures seen with MsDps2 were very similar to those shown for the *E. coli* and *Helicobacter pylori* Dps-DNA nucleoid [15], [16]. These results suggested that the MsDps2 protein is involved in the formation of the nucleoid structures and probably plays a role in the compaction of the *M. smegmatis* nucleoid similar to the *E. coli* Dps protein.

### The N-Terminus of MsDps2 and DNA Condensing Ability

**Figure 3:**
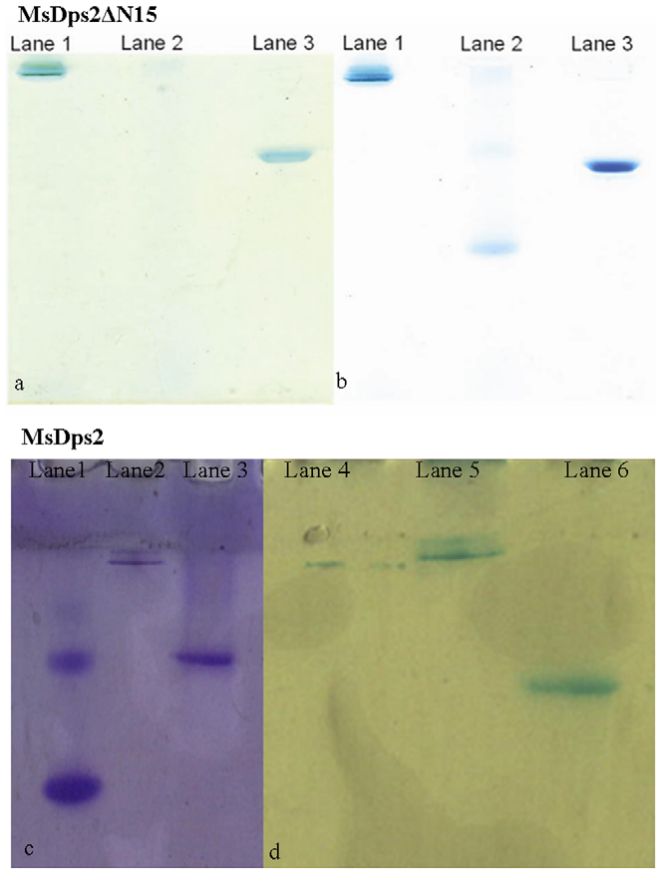
MsDps2ΔN15 has an intact oligomeric structure. Iron-binding assay with the MsDps2ΔN15 shows that the protein accumulates iron like the full-length MsDps2. a) shows the staining for iron by the Prussian blue method. Lane 1: Ferritin; Lane 2: BSA; Lane 3: MsDps2ΔN15. b) shows the same gel stained with Commassie blue. c) MsDps2 protein is seen to accommodate externally added iron in Fe^3+^ state as analysed with potassium ferricynaide method. Here spleen ferritin and BSA were used as positive and negative controls, respectively, on a 10% Native PAGE. d) Subsequent to staining for iron the gel was stained with Coomassie blue (left panel). Lane 1: BSA, Lane 2: Ferritin and Lane 3: MsDps2 protein.

**Figure 4:**
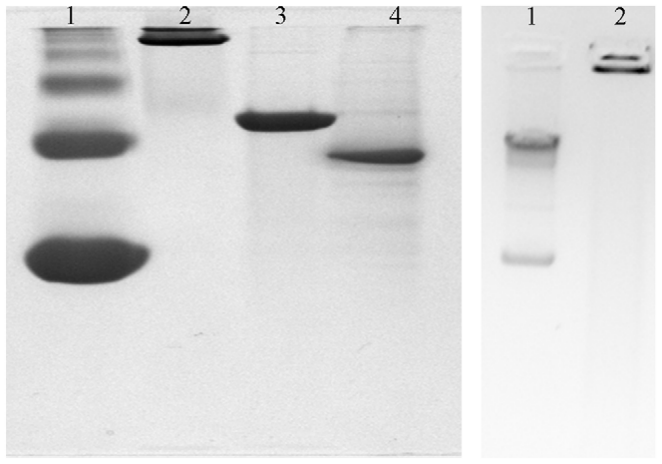
Oligomeric status of MsDps2ΔN15 and its DNA bound complex. **A**) The N-terminal deleted protein MsDps2ΔN15 is a dodecamer like the full-length protein. Lane 1 and 2 are the markers, BSA and Ferritin respectively, on a 10% native gel; Lane 3 is MsDps2; while lane 4 shows MsDps2ΔN15. **B**) MsDps2ΔN15 protein in 20 mM Tris-HCl (pH 7.9), 200 mM NaCl, purified by DE52 ion-exchange chromatography was checked on a 0.8% agarose gel for bound DNA.Lane 1: Free plasmid DNA; Lane 2: DNA+ MsDps2NΔ15 (1∶10^4^ molar ratio).

**Figure 5:**
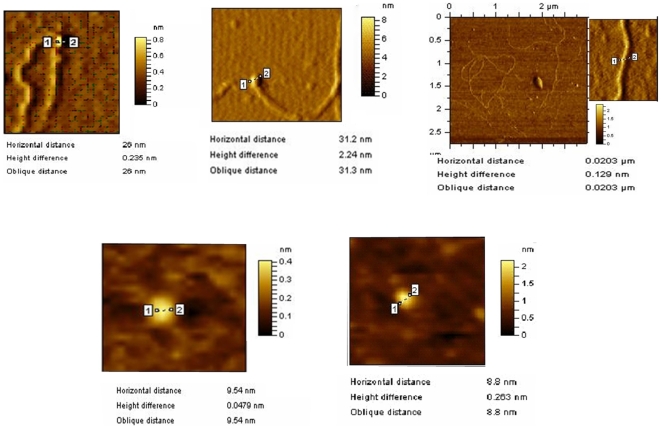
Comparison between the interaction of MsDps2 and MsDps2ΔN15 with DNA by AFM analysis. A) MsDps2ΔN15 forming a complex with DNA. B) MsDps2 forming a complex with DNA. C) Plasmid DNA. D) MsDps2 (without DNA). E) MsDps2ΔN15 (without DNA).

From the sequence alignment of MsDps2 with other Dps proteins, it was apparent that MsDps2 is devoid of any C or N-terminal tails as seen with MsDps1 or the *E.coli* Dps [9]. These extensions confer DNA binding ability to the *E.coli* Dps and MsDps1 proteins [11], [12], [17]. On the contrary, the N-terminus of MsDps2 does not have any characteristic DNA-binding signature and the positively charged residues are distributed evenly throughout the sequence. However, crystal structure analysis showed that the N-terminus of MsDps2 resides at the dodecameric surface, an important prerequisite for DNA-binding. Thus, it is likely to be involved in the DNA binding activity of MsDps2. Additionally, the N-termini from various dodecamers line the intermolecular spaces between the hexagonally closed packed layers of MsDps2 molecules [9]. Interestingly, we observed that the addition of a hexahistidine tag at the N-terminus abrogated the DNA-binding activity of MsDps2 (data not shown). These observations suggested a role for the N-terminal domain of MsDps2 in its DNA-binding ability. In order to test this possibility, we cloned a deleted version of MsDps2 lacking 15 amino acids from the N-terminus, MsDps2ΔN15. The structural integrity of the deleted protein was further supported by its iron-binding activity as shown in figure 3 (panel a and b). For comparison, we have included here the iron binding ability of the intact MsDps2 protein (fig. 3, panel c and d). The protein accumulated iron in its inner cavity like the full-length protein, suggesting that its overall structure is intact. The protein forms dodecamer (fig. 4A), also binds DNA in gel retardation analysis (fig. 4B). AFM analysis of MsDps2ΔN15-DNA complex showed a particle of diameter around 26 nm (Fig. 5A) and corresponds to the DNA-bound protein. This is in contrast to the MsDps2 protein which exists with a larger particle size of 31 nm (Fig. 5B). The difference in the heights (Fig. 5A and 5B) of the DNA-protein complex is also significant. These results indicate that less DNA is bound to MsDps2ΔN15. Thus, N-terminus plays a role in the DNA binding of MsDps2. Free DNA, MsDps2 and MsDps2ΔN15 without DNA has been imaged as controls as shown in figures 5C–E. The values for the diameter of DNA (fig. 5C) and the protein (fig. 5D and E) are within limits. Each measurement has been carried out at least five times with same cursor position. One may note that upon removal of 15 amino acids from the N-terminal, the protein looses its height compared to the native protein, although horizontal distance remains same.

The presence of two independent Dps homologues in *M. smegmatis* suggested that the two proteins performed distinct functions within the cell. The MsDps1 protein is over expressed in the cell under conditions of starvation and osmotic stress at both transcriptional as well as translational level [8], which correlates with its *in vitro* properties of protecting DNA against physical damage and oxidative stress [6]. However, MsDps2 has a more direct role in compaction of DNA and thus its transcriptional regulation will be an important aspect to study.

For this purpose purified antibody against the MsDps2 was generated. The purified antibody was used to detect the expression of MsDps2 in wild type *M. smegmatis* cultures grown under various conditions of growth, which included starvation, very late stationary phase and biofilm growth. MsDps2 protein was not found to be expressed in the lysates under any of the conditions tested (not shown). Although a negative result cannot be taken as a firm conclusion, we would like to suggest that perhaps MsDps2 is not stress regulated and need to be studied further.

We recently have reported that *msdps1* is transcribed by RNA polymerase containing ECF sigma factors [8]. Here, we analyze the transcription complex at the *msdps2* promoter through *in vitro* single round run-off transcription assay. Prior to that, a DNA-protein pull down assay was designed involving the linear biotinylated *msdps2* promoter DNA and an equimolar mixture of reconstituted RNA polymerases containing sigma factors A and B. Similar protocol has been followed as described earlier for biotinylation, immobilization and single round transcription [8].

### The *msdps2* Promoter Is Recognized by Both Principle Sigma Factor A and Principle Like Sigma Factor B Reconstituted RNA Polymerases

**Figure 6:**
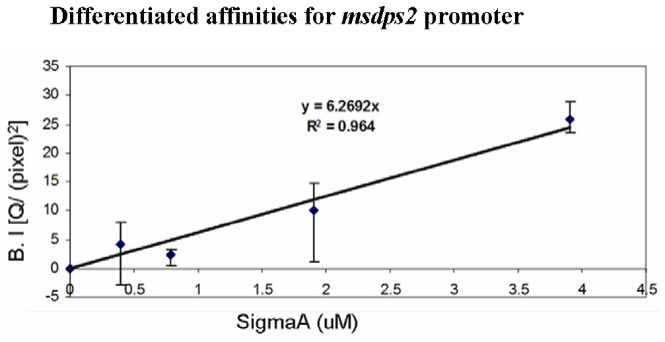
Standard calibration curve using known molar concentrations of pure σA protein from *M. tuberculosis*. The band intensity (B.I) in Y axis represents the relative intensities of the western blots obtained with different concentrations of *M. tuberculosis* σ^A^. In silico analysis was carried out with the Fujifilm Multi-Gauge software. All experiments were performed in triplicates and the average value was taken.

In order to isolate and characterize a transcription complex at *msdps2* promoter, we need to identify the upstream promoter sequence first. However, it was difficult to characterize the same with *in vivo* primer extension method [8], as we do not know under what condition the transcription of *msdps2* is activated. Thus, we cloned a 778 base pair upstream sequence of DNA from the translational initiation site of the *msdps2* gene, in mc^2^155 genomic DNA sequence, assuming that the promoter element will be a part of the same. Preliminary multiple round *in vitro* transcription on this template showed appreciable RNA product with the core RNA polymerase of *M. smegmatis* reconstituted with *M. tuberculosis* σ^A^ and σ^B^ factors (not shown). We have shown before that both *M. tuberculosis* σ^A^ and σ^B^ share significant sequence homology with that of *M. smegmatis* [8]. The DNA fragment was then immobilized on streptavidin coated agarose beads (Sigma Aldrich). We reconstituted *M. smegmatis* core RNA polymerase with different concentration of σ^A^ or σ^B^, named as Eσ^A^ and Eσ^B^ respectively and carried out pull-down experiments and probed with antibodies against the respective sigma factors. The western blots were scanned using Multi Gauge V2.3 software (*in silico*) and the transcript band intensities were quantitated through densitometry. In order to quantitate the differential affinity of the two sigma factors reconstituted holo RNA polymerases namely Eσ^A^ and Eσ^B^ for *msdps2* promoter, a calibration curve was constructed (fig. 6). Table 1 shows the comparative affinity for Eσ^A^ and Eσ^B^ towards *msdps2* promoter. One should note that we have not analyzed the association of any ECF sigma factor reconstituted RNA polymerases with *msdps2* promoter. However, the above experiment has been done to compare the sigma factor specificity of *msdps2* promoter vis-à-vis *msdps1* promoter, where *msdps1* promoter is transcribed by RNA polymerase containing ECF sigma factors only [8]. The presence of any other sigma factors association with *msdps2* promoter, apart from the principle sigma factors, need to be explored.

**Table 1:** Quantification of the band intensities in the western blots, as obtained by probing the eluted fractions from *in vitro* pull-down assay, using Multi-Gauge software.

RNA polymerase (reconstituted)	Concentration of core protein (µM) added during reconstitution	Concentration of sigma proteins (µM) added during reconstitution	Concentration of sigma proteins in the eluted fraction (µM) as obtained from quantitative analysis
*Eσ^A^	0.25	0.5	0.21
	0.95	1.9	0.92
Eσ^B^	0.25	0.5	0.05
	0.95	1.9	0.03

The amounts of core RNA polymerase (*M. smegmatis*) and sigma proteins (*M. tuberculosis*) added during reconstitution were known and the amounts of sigma proteins bound to the *msdps2* promoter were calculated using the standard calibration graph (figure 6).

### Single-Round *In Vitro* Run-Off Transcription Assay

**Figure 7:**
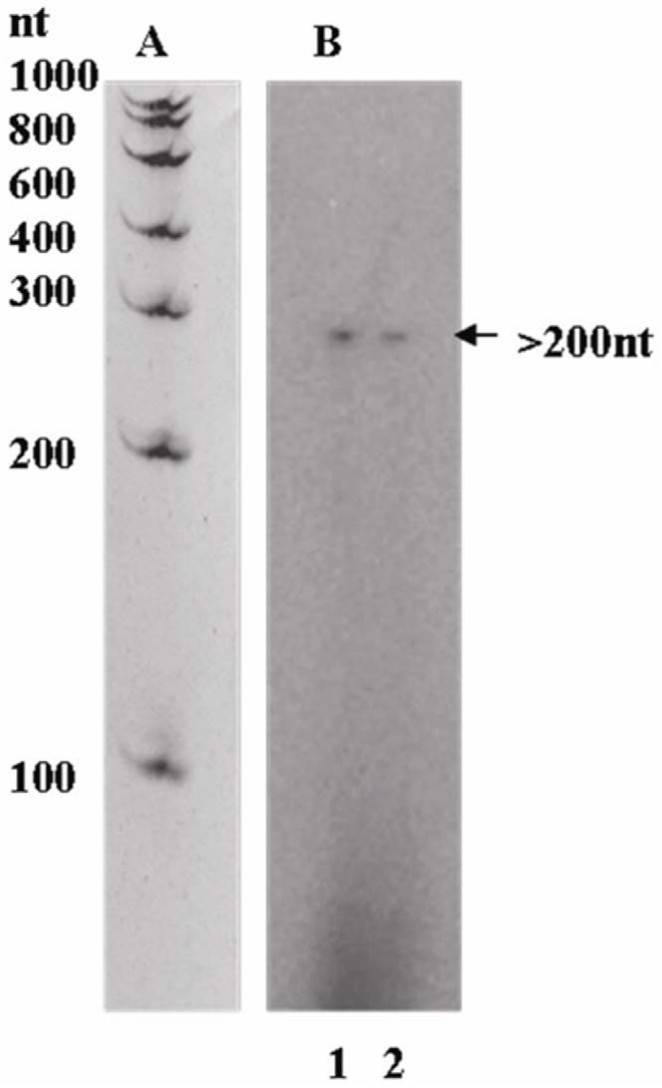
Single-round heparin-resistant run-off transcription at the *msdps2* promoter carried out with *M. smegmatis* reconstituted holo-RNA polymerases. **A**) A 10% polyacrylamide gel containing 6M urea shows an RNA ladder run separately and matched as described before (Chowdhury *et al.,* 2007). **B**) *M. tuberculosis* σ^A^ and σ^B^ were reconstituted with *M. smegmatis* core RNA polymerase. The intensity of each transcript band as obtained from phosphorimager analysis showed the mRNA transcripts for Eσ^A^ (*lane 1*) and Eσ^B^ (*lane 2*).

Reconstitution of holo-RNA polymerases using *M. smegmatis* core (α_2_ββ'ω) and the *M. tuberculosis* σ^A^ and σ^B^ were performed following the same protocol as explained in the previous study. The resulting heterologous polymerases Eσ^A^ and Eσ^B^ were then subjected to single round run-off transcription in the presence of standardized amount of heparin (in order to stop transcription after one round), on a linear 967 bp DNA fragment containing 767 bp upstream *msdps2* promoter region and 200 base pair (bp) of the gene. mRNA transcripts were resolved on a 10% polyacrylamide gel containing 6M urea. Figure 7 panel A shows the RNA ladder ran separately and matched with the test gel [8]. Approximately >200 nucleotide (nt) of transcripts were obtained when the gel was examined in a phosphorimager (Fujifilm) system as a result of incorporation of the radioactive α-P^32^ labeled UTP to the product mRNA (fig. 7) with both Eσ^A^ (Panel B; lane 1) and Eσ^B^ (Panel B; lane 2). We demonstrated before, that no single round transcription takes place with core enzyme alone (data not shown).

### Mycobacterial Dps Proteins Belong to Two Distinct Classes

**Figure 8:**
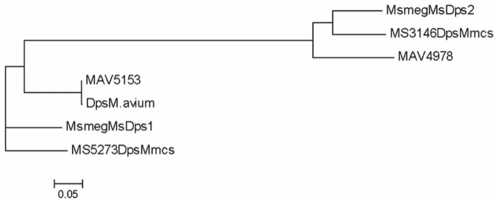
Phylogenetic analysis of mycobacterial MsDps2. Phylogenetic tree of the Dps proteins reveals that MsDps1 and MsDps2 represent two distinct groups among mycobacterial Dps proteins. *M. avium paratuberculosis* has one only Dps which falls into the MsDps1 category.

The presence of two Dps proteins is intriguing, given that it is observed in various species of bacteria. In order to understand the need for two Dps homologues in *M. smegmatis*, we carried out a bioinformatic comparison of the MsDps2 protein with other members of the Dps family. ClustalW analysis was done to compare the sequence of MsDps2 and other Dps family proteins and a phylogenetic tree was constructed based on the sequence analysis. Despite being coded for in the same organism, the MsDps1 and MsDps2 proteins are not very close to each other in sequence identity. This suggests that MsDps2 is not the exact duplicate of MsDps1, sequence-wise, and by corollary, in their functional roles too. Thus the two proteins- MsDps1 and MsDps2 are independent homologues in *M. smegmatis* with possibly unique functions. Additionally, MsDps1 has a long C-terminal tail that contains the DNA-binding motif [10], [11], which is lacking in MsDps2. Upon comparison with Dps homologues from other bacteria, we found that the mycobacterial Dps homologues fall into two groups represented by the MsDps1 and MsDps2 proteins of *M. smegmatis,* respectively. The MsDps1 group proteins from *Mycobacterium smegmatis, Mycobacterium avium* and *Mycobacterium sp.mcs* constitute a separate class from the MsDps2 homologues from the same organisms. Interestingly, there is no Dps homologue in the pathogenic mycobacteria *M. tuberculosis and M. leprae*. Figure 8 shows only the mycobacterial Dps groups. *M. avium paratuberculosis* has only one Dps homologue which belongs to the MsDps1 class, while *M. smegmatis*, *M. avium* and *Mycobacterium sp. mcs* have both the MsDps1 and MsDps2 homologues which are phylogenetically equidistant from each other. Thus, not only are two Dps proteins present in various bacteria, the mycobacterial Dps homologues fall into two distinct categories that are equidistant from each other.

## Discussion

The discovery of a second Dps homologue raised the obvious question, what is the need for the presence of two Dps proteins and what is the role of the second Dps in the physiology of *M. smegmatis*?

Successive attempts to detect MsDps2 protein induction inside the wild type cells, as a function of variable stress factors, failed to show any protein expression. Alternatively, we can look at the promoter region of this gene and try to understand its regulation at the mRNA level.

We show that the MsDps2 protein forms DNA-Dps nucleoid-like structures *in vitro*. A role for the N-terminal 15 amino acids in the DNA-binding property of MsDps2 has been indicated by AFM analysis of an N-terminal deleted version of the protein. The propensity of MsDps2 to bind and compact DNA could be deleterious to the cells and shut down protein synthesis, if left unregulated. The lethal effect of DNA compaction from an artificial overexpression of a nucleoid protein H-NS, in *E.coli* is already documented [18]. At the same time, nucleoid-compaction is one of the mechanisms used in the protection of the cellular genetic material under conditions of stress [19]. Indeed Dps is a starvation and stationary phase specific protein with a role in nucleoid-condensation, as shown in *E.coli* [4]. We suggest that the expression of MsDps2 is tightly controlled *in vivo* to prevent unregulated compaction of the bacterial nucleoid. The association of housekeeping sigma factors A and B to regulate *msdps2* promoter as compared to a stress-specific sigma factors associated with *msdps1* promoter, also endorses the above speculation of a complex regulation of expression of the protein.

## Materials and Methods

Bacterial growth conditions, media composition, cloning, protein purification, MALDI analysis, native gel analysis and gel retardation assay were done as described previously [12]. MsDps2ΔN15 was cloned from the plasmid containing the full-length version of MsDps2 in pET21b using the primers 5′ GGTTTCCATATGACACCGGAG 3′ (forward primer) and 5′ GGCGGTTAGAAGCTT AGACC 3′ (reverse primer) which have *Nde1* and *HindIII* sites, respectively. These sites were used to clone the gene into the *Nde1* and *HindIII* sites in pET21b to create the final plasmid pET*msdps2Δn15*. The MsDps2ΔN15 was also purified like MsDps2 as described previously [9]. For promoter analysis assays, *M. smegmatis* wild-type strain mc^2^155 [20] was grown in MB7H9 medium supplemented with 2% glucose and 0.05% Tween-80.

### Quantification of Band Intensity in GRA Experiments

Multigauge analysis was done to quantify the relative band intensities of the DNA in the wells after the gel retardation assay (GRA). Briefly, 2 µl of pGEM DNA (2.9 kb) was mixed with 18 µl of MsDps2 at a protein: DNA molar ratio of 10^4^:1 in 20 mM Tris–HCl (pH 7.9), 200 mM NaCl, and incubated at 37°C for 30 min. The complex was then resolved on a 0.8% agarose gel in 1X TAE [Tris-acetate–ethylenediaminetetraacetic acid (EDTA)] buffer consisting of 40 mM Tris-acetate, 20 mM sodium acetate and 1 mM EDTA (pH 8.0). The electrophoresis was carried out at a constant voltage of 50 V. The gel was stained with ethidium bromide and observed under UV light. The Dps–DNA complex cannot move into the gel matrix and therefore remained in the well of a 0.8% agarose gel as opposed to the unbound DNA that was visible as a band in the gel (Fig. 1). The well in which the plasmid alone was loaded was taken as the blank. The relative intensities of the DNA in the wells of protein alone and protein with DNA added were quantified with respect to the blank. Finally, the intensity/ (pixel) ^2^ was calculated and represented in the legend of figure 1. The software used for the quantification was Multi-Gauge V 2.3 (Fujifilm). The area (pixel)^ 2^ under each band was kept constant and the intensity values were all normalized with respect to a blank area in the same blot.

### AFM Analysis

AFM analysis was done using the 5500 AFM imaging instrument from Agilent Technologies. Freshly cleaved mica discs were used as substrate on which the DNA and protein samples were immobilized. Prior to the immobilization, plasmid pMV261 (4.5 Kb) was added to 0.5 µg/ml of the protein (MsDps2 or MsDps2ΔN15), in a buffer consisting of 40 mM HEPES pH 7.0, 10 mM MgCl_2_ and 10 mM NiCl_2_. The reaction mixture was then absorbed onto the mica for another 30 minutes. It was then washed three times with 200 µl of MilliQ grade water. Images were captured in a 256/256 and 512/512 pixel format at a speed of 0.5 line/sec. The imaging was done in air, at 22°C. Analysis of the image was done using the PicoImage software.

### Electron Microscopic (EM) Analysis

MsDps2 protein in 20 mM Tris, 200 mM NaCl was placed on copper grids. After two minutes of absorption at room temperature, the sample was negatively stained with uranyl acetate for five minutes. Specimens were examined in a Jeol 100 CxII electron microscope at 80 KV. The photographs were taken at 50,000× magnification. The diameters of the rings were measured from EM negatives with the aid of Wild-Heerbrugg MPS12 zoom stereomicroscope.

### Reconstitution of RNA Polymerase Holoenzymes from *M. tuberculosis* σ^A^ and σ^B^ with Core RNA Polymerase from *M. smegmatis* Followed by *In Vitro* Transcription

The protocol was followed the same way as described previously [8]. *M. tuberculosis* sigma factors σ^A^ and σ^B^ were used for reconstitution. Purified recombinant *M. tuberculosis* sigma proteins A and B were isolated from *E. coli* over-expressing strains.

For *in vitro* transcription assay, σ^A^ and σ^B^ reconstituted RNA polymerases Eσ^A^ and Eσ^B^ were used for either multiple or single round transcription. However, as the +1 transcription start site of the *msdps2* promoter is not known, the expected size of the mRNA transcript in the single round run-off assay cannot be estimated accurately.

### Iron-Binding Assay: Staining of Iron-Binding Proteins

Purified MsDps2ΔN15 and MsDps2 protein in 20 mM Tris-HCl (pH 7.9), 200 mM NaCl, BSA and Ferritin were incubated with 1 mM ferrous sulphate for 1 h at room temperature. The products were resolved on a 10% native PAGE. The gel was then stained with potassium ferricyanide solution (100 mM K_3_(Fe(CN)_6_) in 50 mM Tris-HCl, pH 7.5, 100 mM NaCl) for 10 min in the dark and destained with 10% trichloroacetic acid/methanol solution [21]. After taking an image of the stained gel, it was subjected to Coomassie blue staining using standard techniques. Horse spleen ferritin and BSA were used as positive and negative controls, respectively.

### Bio-Informatic Analysis

Multiple sequence alignment of MsDps2 with other Dps family members was done using MultAlin [22] after selecting for other Dps family members from the TIGR database. Phylogenetic trees were constructed using the ClustalW sequence alignment tool [23] from the EMBL-EBI server and displayed using the MEGA4 software [24]. The group was derived from an analysis of 36 Dps proteins from various bacteria (including the homologues from *E. coli*, *Deinococcus radiodurans*, *Bacillus subtilis* etc) with the average score ranging around 25 for most Dps proteins, while ranging around 75 among members of each of the two mycobacterial Dps groups. Other Dps homologues have not been found to fall into such distinct clusters from the analysis.

### Generation of Antibody against MsDps2 Protein

Polyclonal antibody raised against MsDps2 in rabbit was purified using affinity purification. Briefly, the serum proteins were precipitated with 50% ammonium sulphate and the pellet was washed twice with 50% ammonium sulphate in 100 mM Tris-HCl (pH 7.9). The pellet was then solubilised with 100 mM Tris-HCl (pH 7.9), 100 mM NaCl and dialysed against 1 litre of the same buffer. In order to make an affinity matrix to purify antibody specific to MsDps2 from the serum, the purified MsDps2 protein was desalted by extensive dialysis against a buffer containing 50 mM HEPES-NaOH (pH 7.9). The dialyzed protein was coupled to NHS (N-hydroxysuccinimide)-activated Sepharose Fast Flow 4B column (Amersham Pharmacia Biotech) according to the manufacturer's specifications. The serum IgG was allowed to bind to the affinity tagged column at 4°C for 45 min. The column was then washed with buffer containing 100 mM Tris-HCl (pH 7.9). Specifically bound immunoglobulins were eluted in 1 ml of 200 mM glycine-HCl buffer (pH 2.5) and immediately neutralized with 30 µL of a 2 M solution of Tris base. The collected fractions were then checked for their specificity using western blot analysis. Those fractions containing specific antibodies were pooled and dialysed against 100 mM Tris-HCl (pH 7.9) 100 mM NaCl, and stored at −70°C in aliquots. Titre of the antibody was 1/1500.

## References

[1] Matin A, Auger EA, Blum PH, Schultz JE (1989). Genetic basis of starvation survival in non differentiating bacteria.. Annu Rev Microbiol.

[2] Frenkiel-Krispin D, Minsky A (2006). Nucleoid organization and the maintenance of DNA integrity in *E. coli*, *B. subtilis* and *D. radiodurans*.. J Struct Biol.

[3] Almirón M, Link AJ, Furlong D, Kolter R (1992). A novel DNA-binding protein with regulatory and protective roles in starved *Escherichia coli*.. Genes Dev.

[4] Kim J, Yoshimura SH, Hizume K, Ohniwa RL, Ishihama A (2004). Fundamental structural units of the *Escherichia coli* nucleoid revealed by atomic force microscopy.. Nucleic Acids Res.

[5] Gupta S, Pandit SB, Srinivasan N, Chatterji D (2002). Proteomics analysis of carbon-starved *Mycobacterium smegmatis*: induction of Dps-like protein.. Protein Eng.

[6] Gupta S, Chatterji D (2003). Bimodal protection of DNA by *Mycobacterium smegmatis* DNA-binding protein from stationary phase cells.. J Biol Chem.

[7] Ceci P, Ilari A, Falvo E, Giangiacomo L, Chiancone E (2005). Reassessment of protein stability, DNA binding, and protection of *Mycobacterium smegmatis* Dps.. J Biol Chem.

[8] Chowdhury R, Gupta S, Chatterji D (2007). Identification and characterization of the *dps* promoter of *Mycobacterium smegmatis*: promoter recognition by stress-specific extracytoplasmic function sigma factors σ^H^ and σ^F^. J Bacteriol.

[9] Roy S, Saraswathi R, Chatterji D, Vijayan M (2008). Structural studies on the second *Mycobacterium smegmatis* Dps: invariant and variable features of structure, assembly and function.. J Mol Biol.

[10] Chowdhury RP, Chatterji D (2007). Estimation of Förster's distance between two ends of Dps protein from mycobacteria: Distance heterogeneity as a function of oligomerization and DNA binding.. Biophys Chem.

[11] Roy S, Saraswathi R, Gupta S, Sekar K, Chatterji D (2007). Role of N and C-terminal tails in DNA binding and assembly in Dps: structural studies of *Mycobacterium smegmatis* Dps deletion mutants.. J Mol Biol.

[12] Roy S, Gupta S, Das S, Sekar K, Chatterji D (2004). X-ray analysis of *Mycobacterium smegmatis* Dps and a comparative study involving other Dps and Dps-like molecules.. J Mol Biol.

[13] Ueshima J, Shoji M, Ratnayake DB, Abe K, Yoshida S (2003). Purification, gene cloning, gene expression, and mutants of Dps from the obligate anaerobe *Porphyromonas gingivalis*.. Infect Immun.

[14] Yamamoto Y, Poole LB, Hantgan RR, Kamio Y (2002). An iron-binding protein, Dpr, from Streptococcus mutans prevents iron-dependent hydroxyl radical formation in vitro.. J Bacteriol.

[15] Frenkiel-Krispin D, Ben-Avraham I, Englander J, Shimoni E, Wolf SG (2004). Nucleoid restructuring in stationary-state bacteria.. Mol Microbiol.

[16] Ceci P, Mangiarotti L, Rivetti C, Chiancone E (2007). The neutrophil-activating Dps protein of *Helicobacter pylori*, HP-NAP, adopts a mechanism different from *Escherichia coli* Dps to bind and condense DNA.. Nucleic Acids Res.

[17] Ceci P, Cellai S, Falvo E, Rivetti C, Rossi GL (2004). DNA condensation and self-aggregation of *Escherichia coli* Dps are coupled phenomena related to the properties of the N-terminus.. Nucleic Acids Res.

[18] Spurio R, Dürrenberger M, Falconi M, La Teana A, Pon C (1992). Lethal overproduction of the *Escherichia coli* nucleoid protein H-NS: ultramicroscopic and molecular autopsy.. Mol Gen Genet.

[19] Ishihama A (1999). Modulation of the nucleoid, the transcription apparatus, and the translation machinery in bacteria for stationary phase survival.. Genes Cells.

[20] Snapper SB, Melton RE, Mustafa S, Kieser T, Jacobs WR (1990). Isolation and characterization of efficient plasmid transformation mutants of *Mycobacterium smegmatis*.. Mol Microbiol.

[21] Leong LM, Tan BH, Ho KK (1992). A specific stain for the detection of non-heme iron proteins in polyacrylamide gels.. Anal Biochem.

[22] Corpet F (1988). Multiple sequence alignment with hierarchical clustering.. Nucleic Acids Res.

[23] Thompson J, Higgins D, Gibson T (1994). CLUSTAL W: improving the sensitivity of progressive multiple sequence alignment through sequence weighting, position-specific gap penalties and weight matrix choice.. Nucleic Acids Res.

[24] Kumar S, Nei M, Dudley J, Tamura K (2008). MEGA: a biologist-centric software for evolutionary analysis of DNA and protein sequences.. Brief Bioinformatics.

